# *PAX7* expression in embryonal rhabdomyosarcoma suggests an origin in muscle satellite cells

**DOI:** 10.1038/sj.bjc.6601040

**Published:** 2003-07-15

**Authors:** N Tiffin, R D Williams, J Shipley, K Pritchard-Jones

**Affiliations:** 1 Section of Paediatric Oncology, Institute of Cancer Research, Belmont, Sutton, Surrey SM2 5NG, UK; 2 Molecular Cytogenetics Laboratory, Section of Molecular Carcinogenesis, Institute of Cancer Research, 15 Cotswold Road, Belmont, Sutton, Surrey SM2 5NG, UK

**Keywords:** rhabdomyosarcoma, *PAX3*, *PAX7*, *FOXO1A*, myogenesis, *MET*

## Abstract

Rhabdomyosarcoma (RMS) is a common paediatric soft tissue sarcoma that resembles developing foetal skeletal muscle. Tumours of the alveolar subtype frequently harbour one of two characteristic translocations that juxtapose *PAX3* or *PAX7*, and the forkhead-related gene *FKHR* (*FOXO1A*). The embryonal subtype of RMS is not generally associated with these fusion genes. Here, we have quantified the relative levels of chimaeric and wild-type *PAX* transcripts in various subtypes of RMS (*n*=34) in order to assess the relevance of wild-type *PAX3* and *PAX7* gene expression in these tumours. We found that upregulation of wild-type *PAX3* is independent of the presence of either fusion gene and is unlikely to contribute to tumorigenesis. Most strikingly, upregulated *PAX7* expression is almost entirely restricted to cases without *PAX3*-*FKHR* or *PAX7*-*FKHR* fusion genes and may contribute to tumorigenesis in the absence of chimaeric *PAX* transcription factors. Furthermore, as myogenic satellite cells are known to express *PAX7*, this pattern of *PAX7* expression suggests this cell type as the origin of these tumours. This is corroborated by the detection of *MET* (*c*-*met*) expression, a marker for the myogenic satellite cell lineage, in all RMS samples expressing wild-type *PAX7*.

Rhabdomyosarcoma (RMS) is the most common soft tissue sarcoma of childhood, accounting for up to 8% of all cases of childhood cancer (Cornelison and Wold, 1997). This embryonal muscle cancer resembles normal preinnervated foetal skeletal muscle, both morphologically and in its expression of muscle-specific genes (including the muscle regulatory factor (MRF) family and skeletal muscle-specific structural proteins). There are distinct histological subtypes of RMS that differ in their clinical presentation and behaviour: the embryonal subtype (ERMS) is more common in young children and has a better prognosis, whereas the alveolar subtype (ARMS) is more common in adolescents, is frequently metastatic at diagnosis, and has a worse prognosis. In addition, a rare pleomorphic subtype (PRMS) predominates in adults, among whom RMS is relatively infrequent (Hollowood and Fletcher, 1994).

The histogenesis of RMS is unclear, although it is generally believed to originate from persistent foetal rhabdomyoblasts that have failed to undergo normal differentiation. Studies of expression patterns of myogenic genes have failed to demonstrate any differences in extent of myogenic differentiation between the two main subtypes, ERMS and ARMS. However, comparative genomic hybridisation (CGH) analysis has shown that ERMS frequently exhibits gains or losses of specific whole chromosomes, whereas ARMS is characterised by the presence of regions of genomic amplification (Weber-Hall *et al*, 1996; Gordon *et al*, 2001). The finding of consistent reciprocal chromosomal translocations only in ARMS has suggested a biological basis for the difference in tumour aggressiveness. Alveolar tumours typically harbour one of the two characterised chromosomal translocations that juxtapose *PAX* gene family member *PAX3* or *PAX7* with the Forkhead-related *FKHR (FOXO1A*) gene. The t(2;13)(q35;q14) translocation results in the *PAX3-FKHR* fusion gene, and the less common t(1;13)(p36;q14) produces the *PAX7*-*FKHR* fusion gene. Both translocations generate an in-frame fusion between the undisrupted *PAX* gene DNA-binding domain and the transactivation domain of the *FKHR* gene.

During normal myogenesis, PAX3 is expressed in migrating myoblasts and is believed to inhibit their differentiation until they reach their destination. It has been suggested that dysregulated expression of PAX3, and/or its normal target genes, is involved in RMS tumorigenesis (Bober *et al*, 1994; Goulding *et al*, 1994; Epstein *et al*, 1996). The chimaeric PAX3-FKHR protein has been studied *in vitro* and shown to retain wild-type PAX3 DNA-binding specificity, but with enhanced transcriptional activation. PAX3-FKHR is therefore a more powerful transcription factor than wild-type PAX3. (Fredericks *et al*, 1995; Bennicelli *et al*, 1996; Anderson *et al*, 1999; Merlino and Helman, 1999). PAX3-FKHR transforms chicken embryo fibroblasts, although wild-type PAX3 is unable to do so (Schiedler *et al*, 1996), and PAX3-FKHR inhibits the differentiation of the C_2_C_12_ murine myoblastic cell line to a greater extent than wild-type PAX3 (Epstein *et al*, 1995). This suggests that enhanced PAX3 activity, through increased transcription levels and transcriptional activity of the PAX3-FKHR chimaeric protein, may be contributing to the oncogenic phenotype of RMS. PAX7 expression in normal myogenesis is concurrent with PAX3 expression in myoblasts in the dermomyotome, although PAX7 expression does not persist in migrating myoblasts. It has been shown that the *PAX7*-*FKHR* fusion gene is frequently amplified in ARMS, suggesting that increased gene dosage may be important in controlling its altered function (Barr *et al*, 1996; Weber-Hall *et al*, 1996; Anderson *et al*, 1999; Barr, 1999, 2001; Fitzgerald *et al*, 2000).

PAX3 and PAX7 appear to have a high degree of functional redundancy in normal myogenesis (Mansouri, 1998), although recent work has proposed that expression of PAX7, and not PAX3, is required for the specification and maintenance of myogenic satellite cells, a distinct lineage of myoblastic precursor cells responsible for the postnatal growth, repair and maintenance of skeletal muscle (Seale *et al*, 2000). Expression of PAX3 and PAX7 has previously been detected in RMS cell lines (Barr *et al*, 1999), here we have measured levels of expression of the *PAX* genes and their chimaeric *PAX-FKHR* derivatives in primary RMS samples. The observations that PAX3-FKHR is a more potent transcription factor than PAX3, and that *PAX7*-*FKHR* is frequently amplified in RMS cases, suggest that there may be a ‘dose’ effect of increased PAX3/PAX7 activity in RMS (Ginsberg *et al*, 1998). Hence, in tumours without *PAX3*/*7*-*FKHR* fusion genes, it is possible that mechanisms other than those associated with *PAX3*/7-*FKHR* fusion genes cause increased expression of wild-type *PAX* genes, with the same tumorigenic outcome as expression of the chimaeric proteins. Additionally, levels of *PAX* gene expression may reflect the cellular origin and stage of myogenic differentiation associated with the tumour.

Real-time PCR accurately measures the copy number of a specific mRNA species in a small sample of total RNA (<1 *μ*g), and is useful for analysis of gene expression in primary RMS tumours because samples are seldom large enough for protein analysis. We used this technique to measure mRNA levels for wild-type *PAX3*, *PAX7*, *PAX3*-*FKHR* and *PAX7*-*FKHR* in RMS, to determine whether higher levels of wild-type *PAX3* and *PAX7* are observed in tumours that do not express *PAX3-FKHR* and *PAX7-FKHR* chimaeric transcripts. Expression of a putative target gene for upregulation by PAX3 and PAX3-FKHR, the oncogene *MET*, has also been measured by RT–PCR to determine whether upregulation of wild-type PAX3/7 activity in the absence of *PAX3*/7-*FKHR* fusion genes may be having a downstream effect.

## METHODS AND MATERIALS

### Preparation of cDNA from RMS samples

The RMS samples have been previously characterised and described (Anderson *et al*, 2001). Total RNA was prepared from frozen RMS primary tumour samples and cell lines using Trizol (Life Technologies, Inc., Scotland, UK) according to the manufacturer's instructions. cDNA was synthesised from 1 *μ*g total RNA using reverse transcriptase ‘Superscript’ (Life Technologies) and random hexamers (Life Technologies). To determine cDNA integrity, PCR was used to detect the ubiquitous housekeeping gene *GAPDH* (primer 1: 5′-CGGGAAGCTTGTGATCAATGG-3′, primer 2: 5′-GGCAGTGATGGCATGGACTG-3′, *T*_m_=55°C, [Mg^2+^]=2.0 mM, product size=358 bp, 35 cycles).

### Real-time PCR to detect levels of PAX3, PAX7, PAX3-FKHR and PAX7-FKHR mRNA

A multiplex TaqMan® reaction was used to measure levels of the gene of interest, and of 18S rRNA. The gene of interest was detected using a FAM/TAMRA-labelled probe, and the rRNA internal control was detected using a VIC/TAMRA-labelled probe. The reaction was performed in 25 *μ*l of 1 × TaqMan® Universal PCR Mix (Applied Biosystems, Foster City, CA, USA) containing primers, probes and cDNA template. Primers and probes were designed to amplify across the *PAX-FKHR* breakpoint in the fusion genes, and the equivalent region of the wild-type *PAX3* and *PAX7* genes, and also to amplify across an exon/exon boundary to ensure no genomic DNA would be amplified. Optimal primer and probe concentrations were determined empirically. Table 1

**Table 1:** Primers and probes used to detect *PAX3*, *PAX7*, *PAX3-FKHR* and *PAX7-FKHR* expression by real-time PCR

**Gene**	**Sequence**	**Concentration (nM)**
PAX3	Primer 1: 5′-CACCAGGCATGGATTTTCC-3′	50
	Primer 2: 5′-TTGTCAGGAGTCCCATTACCT-3′	50
	Probe: 5′-CACCATTGGCAATGGCCTCTCA-3′	50
		
PAX7	Primer 1: 5′-CCACAGCTTCTCCAGCTACTCTG-3′	300
	Primer 2: 5′-GGGTTGCCCAAGATGCTC-3′	300
	Probe: 5′-CCGGTCAGCAACGGCCTGTCT-3′	100
		
PAX3-FKHR	Primer 1: 5′-AGGCATGGATTTTCCAGCTATA-3′	50
	Primer 2: 5′-GGGACAGATTATGACGAATTGAATT-3′	300
	Probe: CACCATTGGCAATGGCCTCTCA-3′	50
		
PAX7-FKHR	Primer 1: 5′-TCTGCCTACGGAGCCCG-3′	300
	Primer 2: 5-GGGACAGATTATGACGAATTGAATT-3′	300
	Probe: 5′-CCGGTCAGCAACGGCCTGTCT-3′	100

shows primers and probes used for the reactions. Cycling parameters used were 2 min at 50°C, 10 min at 95°C, and then 15 s at 95°C followed by 1 min at 60°C for 40 cycles. The reactions were carried out on the ABI Prism 7700 DNA sequencer. Relative expression of the gene of interest and 18S rRNA was determined from a standard curve generated from a dilution series of known positive RMS samples. The RD RMS cell line (RDCL) (American Type Cell Culture, Manassas, VA, USA) was used as a positive control for both *PAX3* and *PAX7* expression, as expression of both the genes was readily detectable in this cell line by conventional RT–PCR (35 cycles). RMS cell line Rh30 was used as a positive control for *PAX3-FKHR* expression, and in the absence of known RMS cell lines expressing the *PAX7-FKHR* fusion gene, a primary tumour (sample 6) with a known t(1;13) translocation was used as a positive control for *PAX7-FKHR* expression. Expression of the fusion genes was readily detectable in these positive controls by RT–PCR (35 cycles). cDNA (0.1 *μ*l) was used in each Real-Time PCR reaction, and level of relative expression for each gene of interest in each sample was measured in four independent experiments. Translocation status is shown for each tumour and had been previously determined by a combination of three methods (cytogenetics, interphase fluorescent *in situ* hybridisation and RT–PCR) in a previous study characterising this cohort of samples (Anderson *et al*, 2001).

### Real-time multiplex PCR of 18S rRNA

The TaqMan® ribosomal RNA control reagents (Applied Biosystems) are designed to detect the 18S rRNA gene in a diverse range of eukaryotes. Levels of 18S rRNA were detected in each sample in tandem with detection of the gene of interest, and used as a control for the total amount of cDNA added to the reaction.

### RT–PCR to detect MET in RMS samples

Primers for the *MET* gene were designed to amplify across an exon/exon boundary to prevent amplification of genomic DNA. The primers were chosen to lie in regions of the sequence not showing significant homology with other known gene family members. Primers used were 5′-TGAATACTGCAGACCAATGTGCTAATAGAT-3′ (forward primer) and 5′-TAGTGATAGATACTGTTCCCTTGTAGCTGC-3′ (reverse primer), (Oswel, University of Southampton, UK), *T*_m_=53°C, 40 cycles. A total volume of 25 *μ*l was used for each PCR (0.5 mM dNTPs, 0.5 *μ*M forward and reverse primers, 2.0 mM Mg, 0.1 *μ*l *Taq* polymerase and 1.0 *μ*l cDNA). To confirm specificity of the PCR reactions, the amplified cDNA fragment (240 bp) was digested with restriction enzyme *Hae*III and DNA fragments (147, 93 bp) were resolved on a 2.5% agarose gel.

## RESULTS

Expression of each of the wild-type *PAX3* and *PAX7* genes and the two fusion genes *PAX3-FKHR* and *PAX7-FKHR* was measured by quantitative real-time PCR in 34 primary RMS tumours and in three RMS cell lines. Expression levels were quantified relative to expression in the RD cell line (RDCL) for *PAX3* and *PAX7*, the Rh30 cell line for *PAX3*-*FKHR* and a primary tumour (sample 6) for *PAX7-FKHR*, since no cell line expressing this fusion gene was available. Table 2

**Table 2:** Real-time data for *PAX3, PAX3-FKHR, PAX7* and *PAX7-FKHR*

			**Pax3**		**Pax3FKHR**		**Pax7**		**Pax7FKHR**	
**Sample I.D.**	**Trans**	**Diagnosis**	**Mean**	**s.d.**	**Mean**	**s.d.**	**Mean**	**s.d.**	**Mean**	**s.d.**
6	1 : 13	ARMS	0.28	0.08	0.00	0.00	1.61	0.90	1.00	0.20
8	1 : 13	ARMS	0.00	0.00	0.00	0.00	11.90	19.96	2.08	0.48
7	1 : 13	ARMS	0.00	0.00	0.00	0.00	0.84	0.75	0.75	0.20
										
30	2 : 13	ARMS	0.00	0.00	2.80	0.86	0.00	0.00	0.00	0.00
43	2 : 13	ARMS	0.28	0.13	1.29	0.08	1.84	1.51	0.00	0.00
35	2 : 13	ARMS	0.00	0.00	1.20	0.75	0.00	0.00	0.00	0.00
31	2 : 13	ARMS	0.85	0.27	0.87	0.11	0.00	0.00	0.00	0.00
40	2 : 13	ARMS	1.32	0.05	3.08	0.41	2.43	1.10	0.00	0.00
32	2 : 13	ARMS	1.15	0.15	2.23	0.18	15.67	8.63	0.00	0.00
12	2 : 13	ERMS	0.00	0.00	0.34	0.42	0.00	0.00	0.00	0.00
38	2 : 13	RMS-nos	0.25	0.07	0.52	0.22	0.00	0.00	0.00	0.00
Rh30	2 : 13	Cell Line	0.18	0.07	1.00	0.04	0.00	0.00	0.00	0.00
4	Neither	RMS-nos	1.45	0.58	0.00	0.00	18.04	7.92	0.00	0.00
3	Neither	ARMS	0.00	0.00	0.00	0.00	0.00	0.00	0.00	0.00
110	Neither	ARMS	0.00	0.00	0.00	0.00	3.80	2.51	0.00	0.00
74	Neither	ARMS	0.00	0.00	0.00	0.00	0.00	0.00	0.00	0.00
88	Neither	ARMS	0.02	0.03	0.00	0.00	126.71	51.76	0.00	0.00
61	Neither	ARMS	0.00	0.00	0.00	0.00	501.39	138.76	0.00	0.00
87	Neither	ARMS	0.33	0.17	0.00	0.00	0.00	0.00	0.00	0.00
107	Neither	ARMS	0.03	0.02	0.00	0.00	45.57	17.14	0.00	0.00
										
25	Neither	ERMS	0.00	0.00	0.00	0.00	228.49	111.33	0.00	0.00
85	Neither	ERMS	0.00	0.00	0.00	0.00	51.02	14.35	0.00	0.00
36	Neither	ERMS	1.20	0.22	0.00	0.00	211.67	62.47	0.00	0.00
82	Neither	ERMS	0.02	0.02	0.00	0.00	88.59	45.35	0.00	0.00
53	Neither	ERMS	0.00	0.00	0.00	0.00	40.22	50.43	0.00	0.00
28	Neither	ERMS	0.00	0.00	0.00	0.00	29.59	27.12	0.00	0.00
77	Neither	ERMS	0.00	0.00	0.00	0.00	2.06	1.67	0.00	0.00
79	Neither	ERMS	1.87	0.40	0.00	0.00	110.51	64.14	0.00	0.00
56	Neither	ERMS	0.35	0.17	0.00	0.00	179.96	44.47	0.00	0.00
100	Neither	ERMS	0.08	0.03	0.00	0.00	0.04	0.04	0.00	0.00
98	Neither	ERMS	0.13	0.08	0.00	0.00	0.65	0.67	0.00	0.00
										
HX170	Neither	Cell Line	0.58	0.07	0.00	0.00	0.00	0.00	0.00	0.00
RDCL	Neither	Cell Line	1.00	0.18	0.00	0.00	1.00	0.24	0.00	0.00
										
99	Neither	PRMS	0.00	0.00	0.00	0.00	0.00	0.00	0.00	0.00
97	Neither	PRMS	0.00	0.00	0.00	0.00	0.00	0.00	0.00	0.00
101	Neither	PRMS	0.00	0.00	0.00	0.00	0.00	0.00	0.00	0.00
95	Neither	PRMS	0.00	0.00	0.00	0.00	0.33	0.47	0.00	0.00

**Figure 1:**
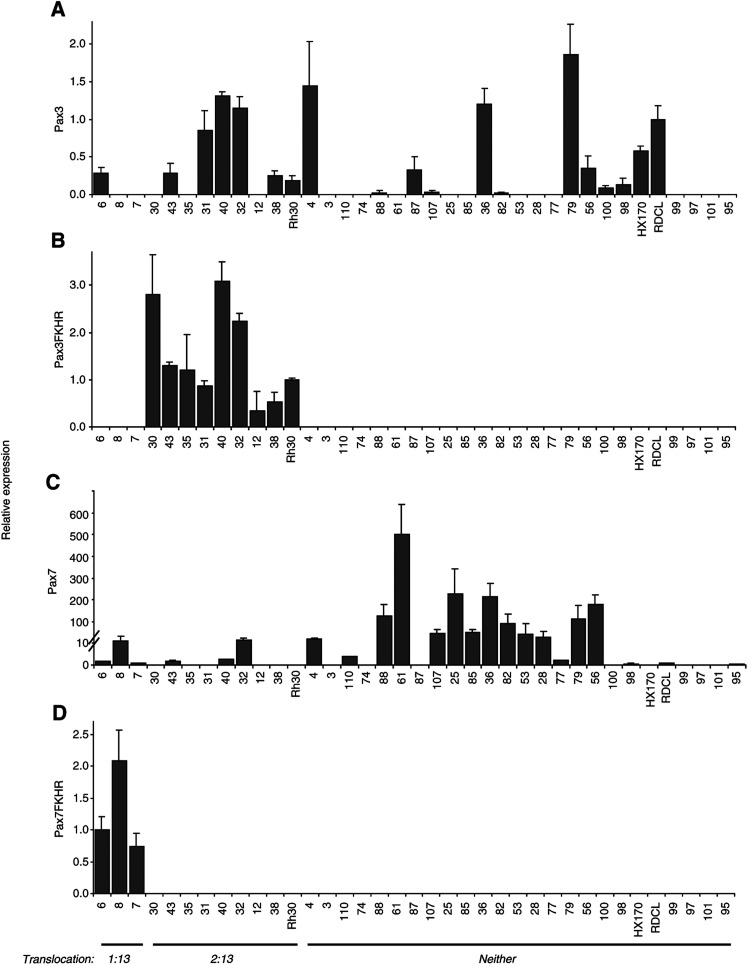
Average relative expression of *PAX3, PAX3-FKHR, PAX7* and *PAX7-FKHR* in RMS samples. (**A**) Expression of *PAX3* is shown relative to the expression of *PAX3* in the cell line RDCL. (**B**) Expression of *PAX3-FKHR* is shown relative to the expression of *PAX3-FKHR* in the Rh30 cell line. (**C**) Expression of *PAX7* is shown relative to the expression of *PAX7* in the cell line RDCL (note different scale due to high levels of expression relative to RDCL=1.0). (**D**) Expression of *PAX7-FKHR* is shown relative to the expression of *PAX7-FKHR* in the RMS sample 6. Translocation status is shown. Error bars indicate standard deviation of four experiments.

Mean values and standard deviations (s.d.) are shown for each gene and each sample. Translocation status (‘Trans’) and histological diagnosis are shown. ARMS=alveolar RMS, ERMS=embryonal RMS, PRMS=pleomorphic RMS, RMS-nos=RMS not otherwise specified. Positive values are shaded.

shows the average expression levels determined in four independent experiments. Since our hypothesis is that the mechanism of enhanced *PAX3* and/or *PAX7* activity will vary according to whether the tumour contains a *PAX3/7-FKHR* fusion gene, tumours are grouped by translocation status rather than histological subtype. To give an overview of how the levels of expression of the four genes relate to each other, relative *PAX3* levels (Figure 1A

) are compared to relative *PAX3-FKHR* levels (Figure 1B), relative *PAX7* levels (Figure 1C) and relative *PAX7-FKHR* levels (Figure 1D).

Among the ERMS and ARMS, there was no clear pattern for wild-type *PAX3* mRNA expression, detected in 16 out of 30 primary ERMS and ARMS tumour samples, nor was there any correlation with the presence of *PAX3/7-FKHR* fusion genes (Figure 1A, B and D). By contrast, *PAX7* mRNA expression corresponded to translocation status rather than histological subtype, with *PAX7* expression detected in both ARMS and ERMS, but mainly restricted to tumours lacking either a *PAX3-FKHR* or a *PAX7-FKHR* fusion gene. *PAX7* expression was detected at lower levels in six out of 11 cases of RMS with *PAX3/7-FKHR* fusion genes, whereas 15 out of 19 ARMS and ERMS cases lacking the fusion genes expressed *PAX7*, generally at much higher levels (Table 2 and Figure 1C). Expression of *PAX7* was readily detectable by real-time PCR and conventional RT–PCR in the control cell line RDCL (no known translocation); however, levels of *PAX7* detected in many of the RMS samples with no known translocation were up to 500 times greater than that of the control cell line (Table 2 and Figure 1C). Adult-type PRMS expressed neither *PAX3* nor *PAX7*.

**Figure 2:**
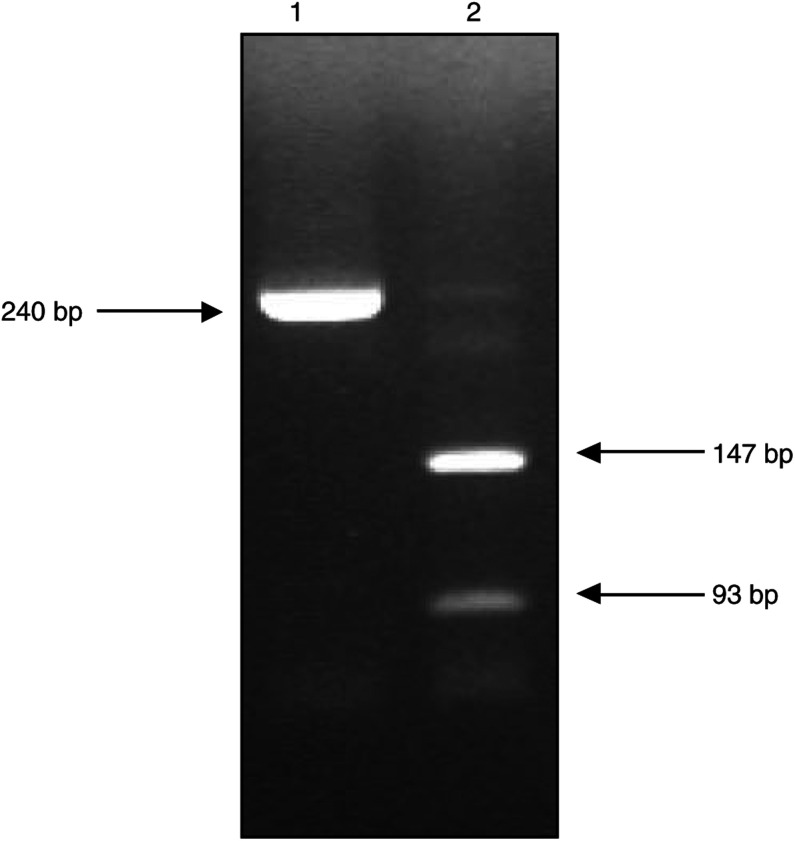
RT–PCR to detect *MET*. Example of *MET* PCR product (240 bp) in lane 1, and restriction enzyme digest products (147, 93 bp) in lane 2.

As expected, expression of the fusion genes was restricted to tumours known to harbour the relevant translocation, with relative expression levels of *PAX3*-*FKHR* varying between 0.3- and 3.1-fold and those for *PAX7-FKHR* between 0.8- and 2.1-fold. Expression of *MET* was detected in 32 out of 37 RMS samples by RT–PCR (Figure 2

). All of the 19 RMS samples with no known translocation and all four of the PRMS tumours were found to express *MET* mRNA. All three of the tumours with *PAX7-FKHR* fusion genes expressed *MET* mRNA, whereas this was not detected in five out of nine samples with the *PAX3*-*FKHR* fusion.

## DISCUSSION

We have investigated whether RMS samples without *PAX-FKHR* fusion genes may have elevated levels of wild-type *PAX3* or *PAX7* expression, which might be implicated in tumorigenesis or indicate cellular origin of the tumour, and have found that elevated *PAX3* expression levels are independent of RMS subtype or presence of a *PAX3/7-FKHR* fusion gene. *PAX3* expression was detected in 16 out of 34 tumour samples, regardless of subtype or translocation status, and it seems unlikely that a ‘dose’ effect of *PAX3* activity may confer a transformed phenotype in RMS in which the *PAX-FKHR* fusion genes do not occur. However, elevated levels of wild-type *PAX7* expression are confined mainly to RMS samples with no known translocation (excluding PRMS cases). *PAX7* and *PAX3* expression levels in these RMS samples are measured relative to expression levels in the RD cell line, which expresses *PAX7* levels comparable to those of normal human myoblasts (Bernasconi *et al*, 1996).

These results agree with data showing *PAX3* and *PAX7* expression in ERMS cell lines published by Barr *et al* (1999) and data from RMS specimens referred to therein. Elevated *PAX7* expression in ERMS cell lines is also reported by Bernasconi *et al* (1996). Here we show greatly elevated levels of *PAX7* expression in a large cohort of primary ERMS tumours, and generally lower, less frequent expression of *PAX7* in primary RMS tumours with *PAX-FKHR* fusion genes. It is possible in ERMS that elevated wild-type *PAX7* expression contributes to transformation in the absence of *PAX3-FKHR* or *PAX7-FKHR* expression, perhaps by suppressing the apoptotic programme that would normally eliminate these cells (Bernasconi *et al*, 1996). *PAX3*, *PAX7* and their chimaeric derivatives were not detected in any of the four PRMS samples included in this study, suggesting that this subtype of RMS is distinct from ERMS and ARMS. This is supported by the distinct histopathology of these tumours and their prevalence in adult rather than paediatric patients (Hollowood and Fletcher, 1994).

PAX7 expression during normal myogenesis is concurrent with PAX3 expression; both genes appear to have the same DNA-binding specificities and a high degree of functional redundancy (Maulbecker and Gruss, 1993; Borycki *et al*, 1999). However, elevated PAX7 expression in ERMS is not accompanied by elevated PAX3 expression, suggesting that this tumour type is not derived from proliferating myoblasts in the dermomyotome. Recent work has shown that expression of PAX7 rather than PAX3 is required for specification and maintenance of myogenic satellite cells (Seale *et al*, 2000). These are a distinct lineage of myoblastic precursor cells responsible for the postnatal growth, repair and maintenance of skeletal muscle (Gussoni *et al*, 1999; Jackson *et al*, 1999; Seale and Rudnicki, 2000). Analysis of *PAX7*^−/−^ transgenic mice implicates PAX7 expression in the specification of myogenic satellite cells from uncommitted side population (SP) stem cell progenitors in skeletal muscle, where PAX7 expression may induce satellite cell specification by restricting alternate developmental programmes (Seale *et al*, 2000). PAX7 expression is an early event during embryogenesis, and satellite cells are not restricted to the somites or limb buds during early embryo development and can move around the embryo (De Angelis *et al*, 1999; Asakura *et al*, 2002). Satellite cells proliferate in response to stimuli such as exercise and injury, and descendents undergo several rounds of cell division before differentiating into new multinucleate muscle fibres (reviewed in Grounds and Yablonka-Reuveni, 1993; Bischoff, 1994; Buckingham, 2001).

PAX7 expression is implicated in the specification and maintenance of satellite cells, and here we have shown that *PAX7* expression is almost completely restricted to RMS tumours with no known translocation. We therefore propose that these tumours may be derived from the myogenic satellite cell lineage. This is consistent with the analysis of *MET* expression by conventional RT–PCR, which shows expression of *MET* mRNA in all 19 tumours with no known translocation: quiescent satellite cells normally express *MET*, the receptor for hepatocyte growth factor, prior to entering S-phase (Cornelison and Wold, 1997). *MET* (c-met) is generally believed to be a downstream target of *PAX3*, and it is unexpected to find that *MET* is only expressed in four out of nine cases of ARMS. However, Epstein *et al* (1996) have previously showed that PAX3 and PAX3-FKHR expression only modestly increases expression of c-met, and that *PAX3-FKHR* is not sufficient in all cases to activate *MET*.

A recent mouse model for RMS has been described by Sharp *et al* (2002) who report that HGF/SF Ink4A/Arf^−/−^ mice develop multifocal sarcomatous malignancies arising from trunk and limb skeletal muscles. In histopathological analysis, the tumours resembled human ERMS, expressing low *Pax3* levels, and elevated *Pax7* levels, which generally mirrored *c-Met* expression. They describe the earlier appearance of hyperplastic satellite cells in the skeletal muscle of these mice, and suggest that these myogenic precursors were the source of the RMS tumours. The authors propose that aberrant activation of the *Pax7/c-Met* pathways may induce formation of ectopic skeletal muscle by encouraging satellite-cell specification at the expense of alternate developmental stem cell programmes.

These data from a novel mouse model of ERMS support our proposal that in cases of human ERMS, elevated *PAX7* expression and consistent *MET* expression indicate an origin in myogenic satellite cells for this subtype of RMS. The inherent on/off proliferative capacity of these cells may increase their susceptibility to transformation, and a distinct cell origin for this subtype of RMS could explain its different behaviour and prognosis. Molecular profiling of myogenic genes in satellite cells and RMS with and without *PAX-FKHR* fusion genes should further define the relation between these cell populations and provide insights into the histogenesis and variable clinical behaviour of the different subtypes of RMS.
